# Supplementary material for: Effects of Insular Cortex on Post-Stroke Dysphagia: A Systematic Review and Meta Analysis
Source: Brain Sci. 2022 Oct 2;12(10):1334. doi: 10.3390/brainsci12101334 (PMC9599629; doi:10.3390/brainsci12101334)
Supplement: Supplementary file 1 [file brainsci-12-01334-s001.zip › brainsci-1925167-supplementary.pdf]

Medline

Search formula

#1 Search (Stroke[Title/Abstract] OR post-stroke[Title/Abstract] OR poststroke[Title/Abstract] OR Hemiplegia[Title/Abstract] OR hemiparesis[Title/Abstract] OR paresis[Title/Abstract] OR paretic[Title/Abstract] OR hemiparetic[Title/Abstract])

#2 Search ((cerebral*[Title/Abstract] OR cerebell*[Title/Abstract] OR brain*[Title/Abstract] OR vertebrobasilar[Title/Abstract])) AND (ischemi*[Title/Abstract] OR infarction[Title/Abstract] OR thrombosis[Title/Abstract] OR embolism[Title/Abstract] OR apoplexy*[Title/Abstract])

#3 Search ((brain*[Title/Abstract] OR cerebral*[Title/Abstract] OR subarachnoid[Title/Abstract])) AND (haemorrhage*[Title/Abstract] OR hemorrhage*[Title/Abstract] OR haematoma*[Title/Abstract] OR hematoma*[Title/Abstract] OR bleed*[Title/Abstract])

#4 Search stroke[MeSH Major Topic]

#5 #1 AND #2 AND #3 AND #4

#6 Search lesion[Title/Abstract] OR site*[Title/Abstract] OR region*[Title/Abstract]

#7 Search dysphagia[Title/Abstract] OR swallow*[Title/Abstract] OR deglutition[Title/Abstract] OR esophageal[Title/Abstract]

#8 #5 AND #6 AND #7

Database	Mesh terms
Medline	**Stroke:** Strokes; Cerebrovascular Accident; Cerebrovascular Accidents; CVA (Cerebrovascular Accident); CVAs (Cerebrovascular Accident); Cerebrovascular Apoplexy; Apoplexy, Cerebrovascular; Vascular Accident, Brain; Brain Vascular Accident; Brain Vascular Accidents; Vascular Accidents, Brain; Cerebrovascular Stroke; Cerebrovascular Strokes; Stroke, Cerebrovascular; Strokes, Cerebrovascular; Apoplexy; Cerebral Stroke; Cerebral Strokes; Stroke, Cerebral; Strokes, Cerebral; Stroke, Acute; Acute Stroke; Acute Strokes; Strokes, Acute; Cerebrovascular Accident, Acute; Acute Cerebrovascular Accident; Acute Cerebrovascular Accidents; Cerebrovascular Accidents, Acute.<br>**Dysphagia:** Deglutition Disorder; Disorders, Deglutition; Swallowing Disorders; Swallowing Disorder; Oropharyngeal Dysphagia; Dysphagia, Oropharyngeal; Esophageal Dysphagia; Dysphagia, Esophageal.
Cochrane library<br>Web of science	**Stroke:** Cerebral Strokes; CVAs (Cerebrovascular Accident); Cerebral Stroke; Strokes, Cerebral; Cerebrovascular Stroke; Strokes, Cerebrovascular; Apoplexy; Stroke, Cerebral; Cerebrovascular Accident; Strokes; Vascular Accidents, Brain; Vascular Accident, Brain; Stroke, Cerebrovascular; Cerebrovascular Strokes; CVA (Cerebrovascular Accident); Brain Vascular Accident; Apoplexy, Cerebrovascular; Brain Vascular Accidents; Cerebrovascular Apoplexy; Cerebrovascular Accidents; Cerebrovascular Accidents, Acute; Strokes, Acute; Acute Stroke; Acute Cerebrovascular Accidents; Stroke, Acute; Cerebrovascular Accident, Acute; Acute Cerebrovascular Accident; Acute Strokes.<br>**Dysphagia:** Dysphagia; Oropharyngeal; Oropharyngeal Dysphagia; Swallowing Disorders; Swallowing Disorder; Disorders, Deglutition; Dysphagia; Deglutition Disorder; Dysphagia, Esophageal; Esophageal Dysphagia.
	**Stroke:** accident, cerebrovascular; acute cerebrovascular lesion; acute focal cerebral vasculopathy; acute stroke; apoplectic stroke; apoplexia; apoplexy; blood flow disturbance, brain; brain accident;

Embase	brain attack; brain blood flow disturbance; brain insult; brain insultus; brain vascular accident; cerebral apoplexia; cerebral insult; cerebral stroke; cerebral vascular accident; cerebral vascular insufficiency; cerebrovascular accident; cerebrovascular arrest; cerebrovascular failure; cerebrovascular injury; cerebrovascular insufficiency; cerebrovascular insult; cerebrum vascular accident; cryptogenic stroke; CVA; insultus cerebralis; ischaemic seizure; ischemic seizure; stroke; thrombotic stroke.<br>**Dysphagia:** aphagopraxia; deglutition difficulty; deglutition disorder; deglutition disorders; difficult deglutition; difficulty in swallowing; difficulty swallowing; dysphagias; swallowing difficult; swallowing difficultness; swallowing difficulty; swallowing disorder.
